# The Fab Fragment of a Humanized Anti-Toll Like Receptor 4 (TLR4) Monoclonal Antibody Reduces the Lipopolysaccharide Response via TLR4 in Mouse Macrophages

**DOI:** 10.3390/ijms161025502

**Published:** 2015-10-23

**Authors:** Binggang Cai, Maorong Wang, Xuhui Zhu, Jing Xu, Wenkai Zheng, Yiqing Zhang, Feng Zheng, Zhenqing Feng, Jin Zhu

**Affiliations:** 1 Department of Infectious Disease, Anhui Medical University Affiliated with Bayi Clinical College, Hefei 230000, China; E-Mail: cbg1021@163.com; 2 Institute of Liver Disease, Nanjing Jingdu Hospital, Nanjing 210002, China; E-Mail: xujingjerry@126.com; 3 Department of Microbiology, Huadong Medical Institute of Biotechnology, Nanjing 210002, China; E-Mails: xh_zhu@163.com (X.Z.); zhengf82@gmail.com (F.Z.); 4 Department of Pathology, Key Laboratory of Antibody Technique of the Ministry of Health, NJMU, Nanjing 210029, China; E-Mails: zwk1987617@163.com (W.Z.); rachel_zhang2005@163.com (Y.Z.); fengzhenqing@njmu.edu.cn (Z.F.)

**Keywords:** humanized anti-Toll like receptor 4 antibody, Fab fragment, lipopolysaccharide, Toll-like receptor 4 signaling

## Abstract

Lipopolysaccharides (LPS) can induce acute inflammation, sepsis, or chronic inflammatory disorders through the Toll receptor 4 (TLR4) signaling pathway. The TLR4/MD2 (myeloid differentiation protein 2) complex plays a major role in the immune response to LPS. However, there is not a good method to suppress the immune response induced by LPS via this complex in macrophages. In this article, we aimed to evaluate the effects of humanized anti-TLR4 monoclonal antibodies on LPS-induced responses in mouse macrophages. The peritoneal macrophages of mice were incubated with anti-TLR4 monoclonal antibodies and stimulated with LPS. The expression levels of cytokines were analyzed by quantitative polymerase chain reaction and enzyme-linked immunosorbent assays. Additionally, activation of various signaling pathways was evaluated by Western blotting. The results showed that the humanized anti-TLR4 monoclonal antibody blocked the inflammatory cytokines expression at both the mRNA and protein level. We also found that the Fab fragment significantly inhibited the nuclear factor kappaB signaling pathway by reducing the phosphorylation of the inhibitor of kappaBalpha and decreasing the translocation of p65, resulting in the suppression of p38, extracellular signal-regulated kinase 1/2, c-Jun N-terminal kinase 1/2, and IFN-β regulatory factor 3 phosphorylation. Therefore, our study showed that this humanized anti-TLR4 monoclonal antibody could effectively protect against LPS-induced responses by blocking the TLR4 signaling pathway in mouse peritoneal macrophages.

## 1. Introduction

Toll-like receptor 4 (TLR4), the human Toll homolog originally known as hToll [1], is one of the first members of the TLR family and has been well characterized as a pattern-recognition receptor (PPR) [2]. TLRs can recognize pathogen-associated molecular patterns (PAMPs) or endogenously sourced damage-associated molecular pattern molecules (DAMPs) produced by the pathogen, resulting in the activation of innate immune responses. The secretion of pro-inflammatory cytokines and type I interferons (IFNs) could be induced after the activation of innate immune responses, eventually removing the invading pathogenic microorganisms [3,4]. TLR4 is mainly expressed in antigen-presenting cells (APCs), including macrophages, monocytes, and dendritic cells (DCs) [5,6]. TLR4 is different from other TLRs in many ways. For example, TLR4 can activate both myeloid differentiation factor 88 (MyD88)-dependent signaling pathways, which induce genes encoding pro-inflammatory molecules, and the expression of type I IFNs through the TIR (Toll/IL-1 receptor) -domain-containing adaptor-inducing IFN (TIRF) signaling pathway [7]. TLR4 is responsible for the recognition of lipopolysaccharides (LPS, endotoxin), the major structural component of the outer wall of gram-negative bacteria, and promotes the immune response against invading gram-negative bacteria. LPS combines with LPS-binding protein (LBP) and then binds with CD14 to form the LPS-LBP-CD14 complex. This complex then interacts with the TLR4/MD2 complex to trigger downstream signaling pathways and activate the inflammatory response [8]. Additionally, LPS can interact with TLR4; this will cause over-activation of TLR4 and subsequently induce the systemic release of pro-inflammatory cytokines, resulting in acute sepsis or chronic inflammation diseases [9]. Therefore, it is important to inhibit the inflammatory response induced by LPS. Although many researchers have attempted to eliminate or neutralize endotoxins using bactericidal proteins, anti-LPS antibodies (or anti-LipidA antibodies), and antimicrobial proteins/peptides, these treatments have not had sufficient beneficial effects.

Monoclonal antibodies (mAbs) are the basis of many different medicines used to treat patients with inflammation-based diseases. In our previous work, we successfully used monoclonal antibody technology to prepare a mouse anti-human TLR4 monoclonal antibody and found that this antibody significantly inhibited the LPS-stimulated generation of tumor necrosis factor (TNF)-α. However, humans can produce human anti-mouse antibodies (HAMAs) against mouse-derived antibodies, resulting in a strong immunological reaction and limiting the clinical applications of this new antibody. Therefore, using phage antibody library technology, we previously amplified humanized anti-TLR4 Fab gene splicing fragments and successfully built an antibody with a humanized anti-TLR4 Fab prokaryotic expression vector, yielding purified anti-TLR4 Fab antibodies.

In this study, we aimed to improve our understanding of this new humanized anti-TLR4 antibody Fab fragment by examining the effects of this antibody on inflammatory cells (specifically mouse macrophages) and TLR4 signaling.

## 2. Results

### 2.1. Purification of the Humanized Anti-TLR4 Antibody Fab Fragment

**Figure 1:**
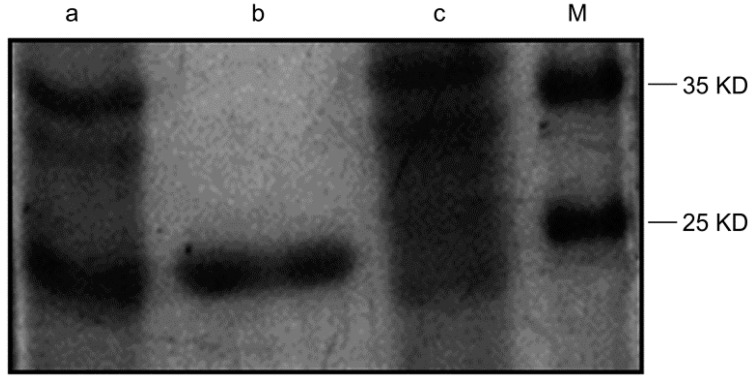
Detection of the purified antibody by plyacrylamide gelelectrophoresis. Lane a: lysate of induced recombinant vector; lane b: the purified Fab; lane c: supernatant of sonicated lysate after purification; lane M: protein marker.

We used a 1 mL His-trap Lambda Fab Select column to purify the recombinant protein. After purification, the majority of the target protein was absorbed by the column and eluted with 0.1 mL sodium acetate solution (pH 2.5). The purification of the antibody was analyzed using SDS-PAGE and colloidal Coomassie brilliant blue staining. A single band was detected on the gel, representing the purified protein (Figure 1).

### 2.2. Specific Binding of the Humanized Anti-TLR4 Antibody Fab with TLR4

**Figure 2:**
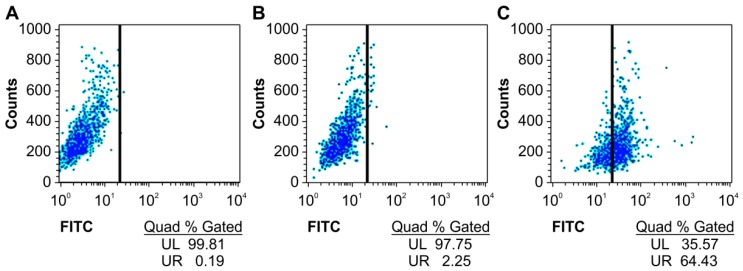
Affinity of anti-TLR4 antibody Fab to TLR4 measured by flow cytometry. (**A**) Blank; (**B**) Without Fab antibody treatment group; (**C**) Fab antibody treatment group. UL: upper left; UR: upper right; FITC: fluorescein isothiocyanate.

We first detected whether the humanized anti-TLR4 antibody Fab fragment could bind to TLR4 expressed on the surface of mouse macrophages using flow cytometry. The specific binding of the antibody with TLR4 was increased by more than 60% compared with that in untreated groups (Figure 2).

### 2.3. Optimization of the Appropriate Concentration of Humanized Anti-TLR4 Antibody Fab for Inhibition of Lipopolysaccharides (LPS)-Stimulated Mouse Macrophages

**Figure 3:**
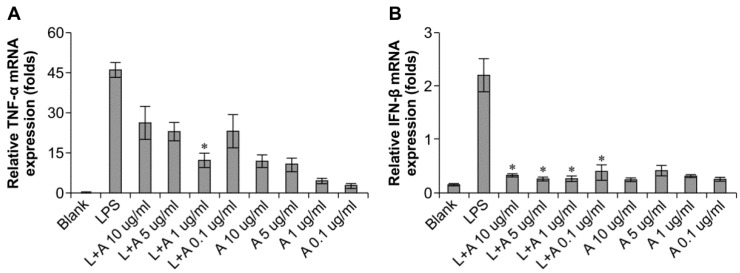

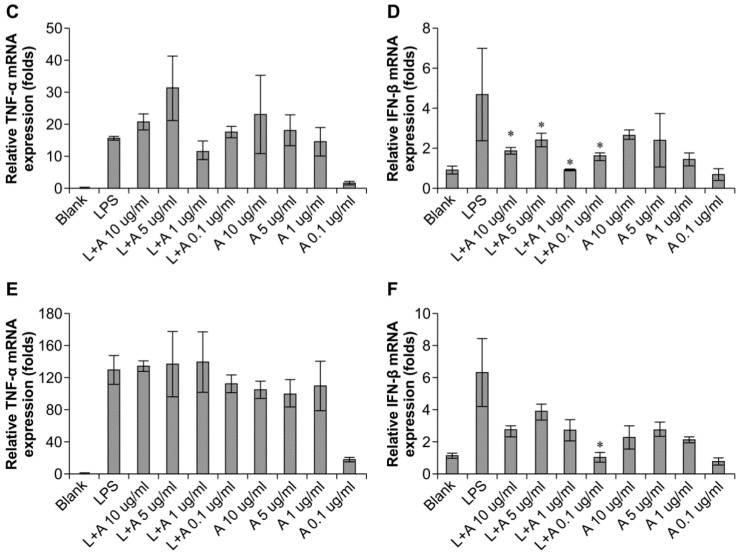
Concentration of humanized anti-TLR4 antibody Fab with effective inhibitory effect on lipopolysaccharides (LPS)-stimulated macrophages. (**A**,**B**) Effects of different concentration of the antibody on inhibition of TNF-α (**A**) and IFN-β (**B**) expression induced by LPS (1 µg/mL) in mouse macrophages; (**C**,**D**) Effects of different concentration of the antibody on inhibition of TNF-α (**C**) and IFN-β (**D**) expression induced by LPS (100 ng/mL) in mouse macrophages; (**E**,**F**) Effects of different concentration of the antibody on inhibition of TNF-α (**E**) and IFN-β (**F**) expression induced by LPS (10 ng/mL) in mouse macrophages. The expressions of TNF-α, IFN-β were measured by Q-PCR. L: LPS; A: humanized anti-TLR4 antibody. Data are shown as mean ± SD of pooled results of three independent experiments (* *p* < 0.05 compared to LPS group).

We then determined the appropriate concentration of the humanized anti-TLR4 antibody Fab to inhibit LPS-stimulated macrophages. The data (Figure 3) showed that the effects of LPS were inhibited in a concentration-dependent manner. When LPS was used at 10 and 100 ng/mL, the humanized anti-TLR4 antibody had similar inhibitory effects on IFN-β expression, regardless of the concentration of antibody; IFN-β expression was reduced to about 50%. In contrast, TNF-α expression was not inhibited by the anti-TLR4 antibody. When the concentration of LPS was 1 μg/mL, treatment with the humanized anti-TLR4 antibody Fab fragment blocked IFN-β expression by 80%–90%. However, at this concentration of LPS, the effects of the humanized anti-TLR4 antibody Fab on TNF-α expression varied according to the concentration of the antibody; approximately 50% inhibition was observed at lower concentrations of the antibody, whereas up to 70% inhibition of TNF-α expression was observed when cells were treated with anti-TLR4 antibody Fab (1 µg/mL). Therefore, these data demonstrated that the optimal results were achieved when using LPS (1 µg/mL) and humanized anti-TLR4 antibody Fab (1 μg/mL).

### 2.4. Inhibitory Effects of the Humanized Anti-TLR4 Antibody Fab on Cytokine Expression after LPS Stimulation of Mouse Macrophages

**Figure 4:**
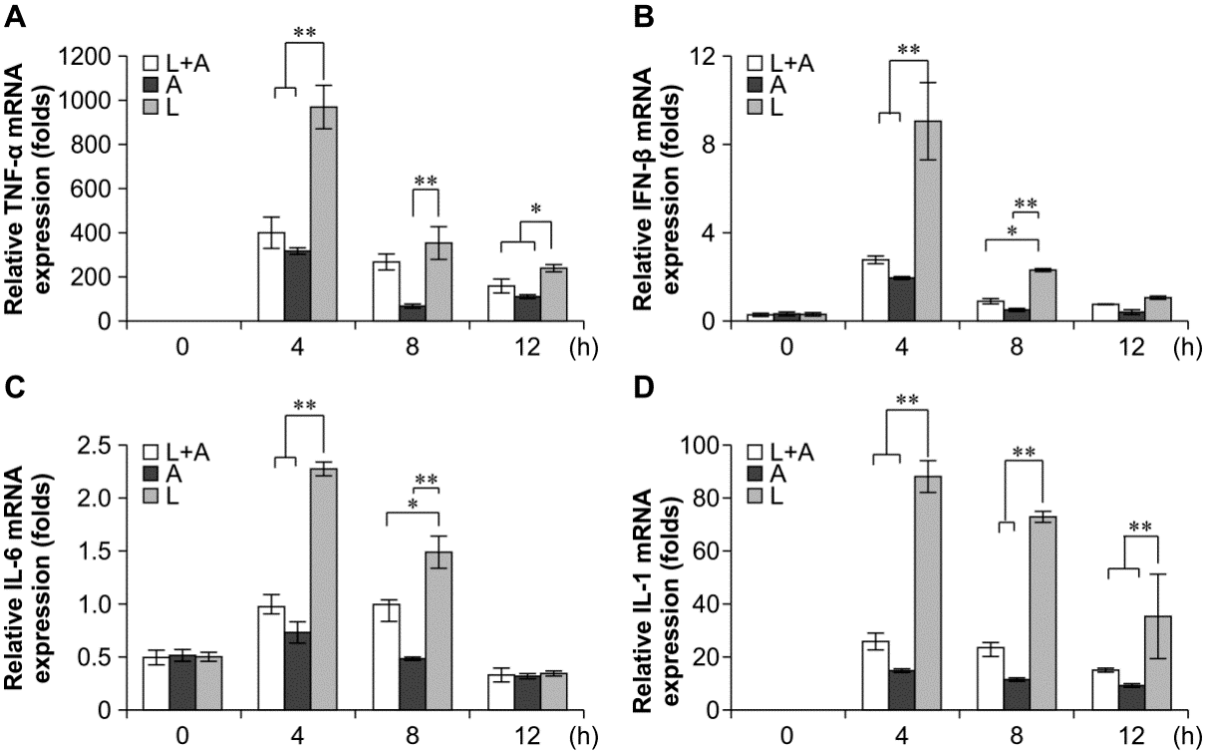
Inhibitory effect of Fab on cytokine transcription in LPS-stimulated mouse macrophages. Humanized anti-TLR4 antibody Fab inhibits the cytokine transcription, including TNF-α (**A**); IFN-β (**B**); IL-6 (**C**); IL-1 (**D**), in mouse macrophages stimulated by LPS. Mouse macrophages were treated with humanized anti-TLR4 antibody for 2 h, and then stimulated with 1 µg/mL LPS for 4, 8, or 12 h. After stimulation the cells were collected, and TNF-α, IFN-β, IL-1, IL-6 were measured by Q-PCR. L: LPS; A: humanized anti-TLR4 antibody. Data are shown as mean ± SD (*n* = 6, * *p* < 0.05, ** *p* < 0.01 compared to LPS group).

To confirm that the release of LPS-induced inflammatory cytokines could be inhibited by the humanized anti-TLR4 antibody in mouse macrophages, we examined the production of mRNAs encoding interleukin (IL)-6, TNF-α, IL-1, and IFN-β by quantitative polymerase chain reaction (qPCR). The results showed that the IL-6, TNF-α, IL-1, and IFN-β expression levels were significantly increased in LPS-induced mouse macrophages as compared with those in the untreated control. Conversely, these markers were decreased following pretreatment with the humanized anti-TLR4 antibody Fab (Figure 4). The inhibitory effects of the humanized anti-TLR4 antibody Fab were strongest at 4 h, reaching 60%–70%. The effects then gradually decreased over time, returning to nearly baseline levels at 12 h.

### 2.5. Inhibitory Effects of the Humanized Anti-TLR4 Antibody Fab on Cytokine Expression in Cell Culture Supernatants after LPS Stimulation of Mouse Macrophages

**Figure 5:**
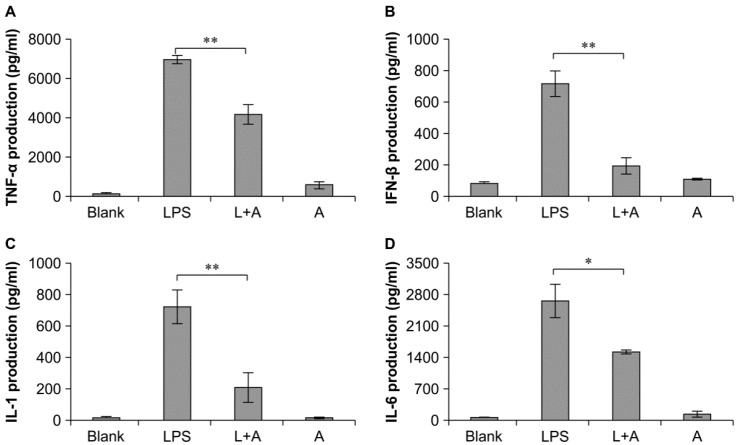
Inhibitory effect of humanized anti-TLR4 antibody Fab on cytokine expression in cell culture supernatant of LPS-stimulated mouse macrophages. (**A**–**D**) represent levels of TNF-α, IFN-β, IL-1, IL-6 in mouse macrophage culture supernatant determined by ELISA analysis. L: LPS; A: humanized anti-TLR4 antibody. Data are shown as mean ± SD (*n* = 3, * *p* < 0.05, ** *p* < 0.01 compare to LPS group).

Next, we analyzed the expression levels of cytokines in cell culture supernatants after treatment with humanized anti-TLR4 antibody Fab. Enzyme-linked immunosorbent assays showed that the levels of TNF-α, IFN-β, IL-1, and IL-6 were significantly decreased following treatment with humanized anti-TLR4 antibody Fab compared with that in the LPS-only group (Figure 5). Moreover, the expression levels of these four cytokines were significantly increased following treatment with LPS or the antibody as compared with that in untreated cells. Therefore, our data showed that humanized anti-TLR4 antibody Fab could inhibit the activation of inflammatory cytokines induced by LPS in mouse macrophages.

### 2.6. Inhibition of LPS-Induced TLR4 Signaling Using the Humanized Anti-TLR4 Antibody Fab

**Figure 6:**
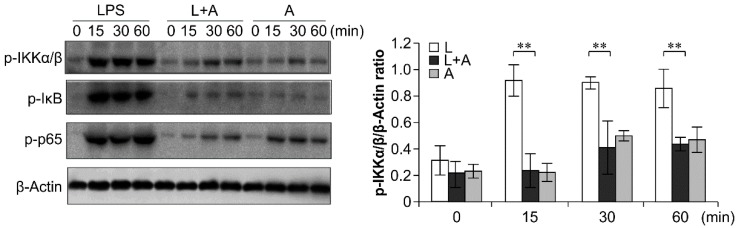

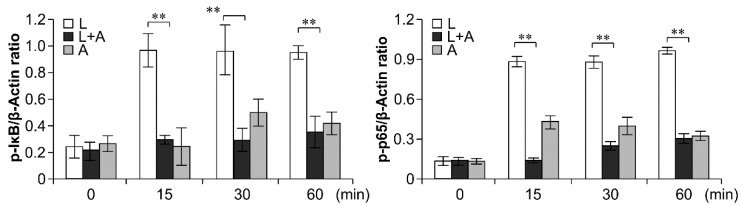
Western blot analysis for inhibition of LPS-induced NF-κB activation by humanized anti-TLR4 Fab. Cells were pretreated with humanized anti-TLR4 antibody for 2 h and further incubated in presence or absence of LPS (1 µg/mL). After immunoblotting, the phosphorylation levels of IKK, IκB, p65 were identified using phosphor-specific antibodies. β-Actin was used to ensure equal loading. L: LPS, A: humanized anti-TLR4 antibody. Data are shown as mean ± SD (*n* = 3, ** *p* < 0.01 compare to LPS group).

**Figure 7:**
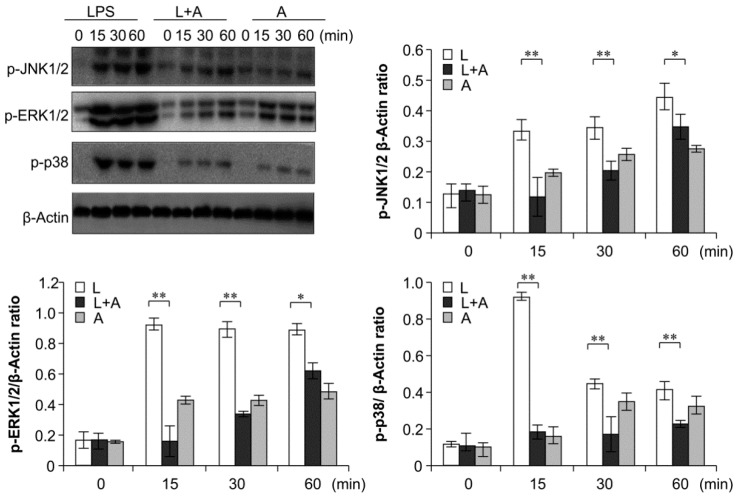
Western blot analysis for inhibition of MAPK activation by humanized anti-TLR4 antibody Fab. Cells were pretreated with humanized anti-TLR4 antibody for 2 h and further incubated in presence or absence of LPS (1 µg/mL). After immunoblotting, the phosphorylation levels of ERK1/2, JNK1/2, p38 were identified using phosphor-specific antibodies. β-Actin was used to ensure equal loading. L: LPS, A: humanized anti-TLR4 antibody. Data are shown as mean ± SD (*n* = 3, * *p* < 0.05, ** *p* < 0.01 *versus* LPS group).

**Figure 8:**
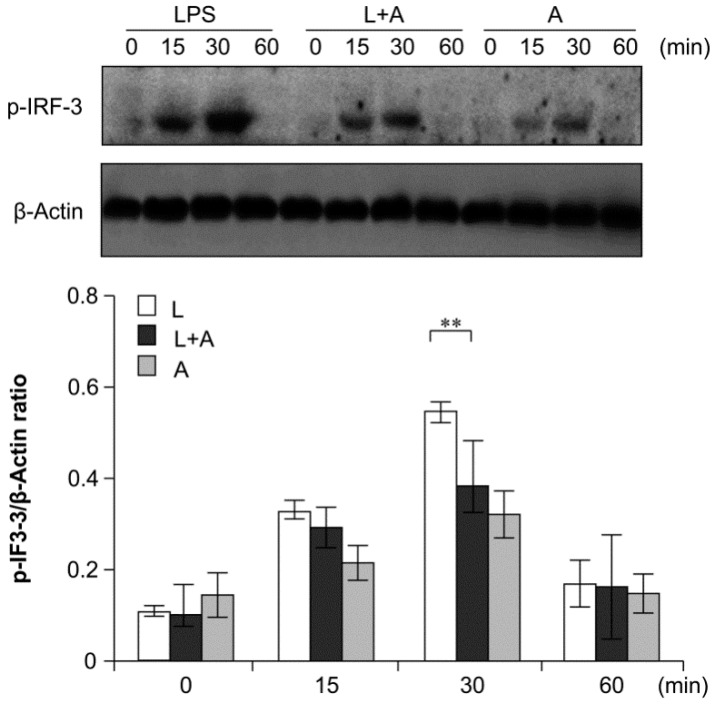
Western blot analysis for inhibition of IRF-3 activation by humanized anti-TLR4 antibody Fab. Cells were pretreated with humanized anti-TLR4 antibody for 2 h and further incubated in presence or absence of LPS (1 µg/mL). After immunoblotting, the phosphorylation level of IRF-3 was identified using phosphor-specific antibody. β-Actin was used to ensure equal loading. L: LPS, A: humanized anti-TLR4 antibody. Data are shown as mean ± SD (*n* = 3, * *p* < 0.05, ** *p* < 0.01 *versus* LPS group).

To elucidate the inhibitory effects of humanized anti-TLR4 antibody Fab on TLR4 signal transduction induced by LPS stimulation, we examined the phosphorylation levels of the nuclear factor κB (NF-κB) signaling pathway, the mitogen-activated protein kinase (MAPK) signaling pathway, and IFN regulatory factor 3 (IRF-3), which function downstream of TLR4. As shown in Figure 6, Figure 7 and Figure 8, LPS stimulation induced significant increases in the phosphorylation levels of these proteins in mouse macrophages; this effect was abrogated by pretreatment with 1 μg/mL humanized anti-TLR4 antibody Fab.

## 3. Discussion

Many studies have examined the role of LPS/TLR4-induced inflammation, and the inhibition of TLR4 using small molecule inhibitors and antibodies is being evaluated for the treatment of various inflammatory conditions in preclinical trials. Various plant-derived drugs, such as wogonoside and celastrol, have also shown promising results against TLR4-dependent LPS-induced inflammation in preclinical drug testing [10]. Therefore, in this study, we aimed to determine the biological functions of our new humanized anti-TLR4 antibody Fab in LPS-stimulated mouse macrophages in preparation for subsequent *in vivo* experiments. Our results showed that the LPS-induced increase in the expression and secretion of pro-inflammatory cytokines was inhibited by pretreatment with humanized anti-TLR4 antibody Fab.

TLRs recognize pathogens and trigger innate immune responses [11], and the TLR4 signaling pathway has been well studied owing to its central role in innate and adaptive immune responses [12]. Activation of TLR4 signaling in mouse macrophages by LPS stimulates NF-κB, MAPK, and IRF3 pathways, leading to the production of inflammatory cytokines [13,14]. In our study, we observed increased phosphorylation of the NF-κB signaling pathway, the MAPK signaling pathway, and IRF3 following LPS stimulation; these effects were blocked by pretreatment with humanized anti-TLR4 antibody Fab. This could explain the decreased expression of the pro-inflammatory cytokines. Moreover, pretreatment with the humanized anti-TLR4 antibody Fab prevented the interaction between LPS and TLR4, which may explain these anti-inflammatory effects observed in mouse macrophages. However, the phosphorylation levels of NF-κB, MAPK, IRF3 signaling pathways and other cytokines also increased following pretreatment with the humanized anti-TLR4 antibody in the absence of LPS. Therefore, we expect that other mechanisms may exist for stimulation of the TLR4 signaling pathway.

TLR4 signaling involves MyD88-dependent and MyD88-independent (TRIF-dependent) pathways [15]. In the MyD88-dependent pathway, MyD88 recruits IRAK-4 and activates TRAF6. TRAF6 then activates TAK1, causing the activation of NF-κB and MAPK pathways, both of which control the expression of pro-inflammatory cytokines. In the MyD88-independent pathway, TRIF recruits TRAF3 and RIP1 to activate IRF3 and NF-κB. IRF3, together with NF-κB, activates the target genes transcription, such as type I IFN [7,16]. Interestingly, we observed greater effects of the antibody on the inhibition of IFN-β expression than on the inhibition of TNF-α, IL-1, and IL-6 in both qPCR analysis and ELISAs. Therefore, it is possible that our humanized anti-TLR4 antibody Fab could block both the MyD88-dependent pathway and MyD88-independent pathway of TLR4 signaling, with greater inhibition of the MyD88-independent pathway.

## 4. Experimental Section

### 4.1. Reagents and Mice

LPS (*Escherichiacoli* 055:B5) was purchased from Sigma (St. Louis, MO, USA). RPMI-1640 medium for cell culture and fetal bovine serum (FBS) were obtained from Gibco (Carlsbad, CA, USA). Dehydration thiol acetate was obtained from Sigma. C57BL/6J mice (6–8 weeks of age) were obtained from SLAC Laboratory Animal Company (Shanghai, China). All animal experiments were carried out in accordance with the National Institutes of Health Guide for the Care and Use of Laboratory Animals. Antibodies targeting phosphorylated ERK1/2, JNK1/2, p38, IκBα, phosphorylated IKKα/β, and phosphorylated p65 were obtained from Cell Signaling Technology (Danvers, MA, USA).

### 4.2. Cells and Cell Culture

Thioglycolate-induced mouse primary peritoneal macrophages were harvested 3–4 days after intraperitoneal injection of thioglycolate broth into C57BL/6J mice, as described previously [17]. Mouse macrophages were cultured in RPMI-1640 medium containing 10% heat-inactivated FBS, 1% antibiotic-antimycotic, and 10 mM HEPES buffer.

### 4.3. Flow Cytometry Analysis

Mouse macrophages (50 μL of 1 × 10^6^ cells/mL) were treated with humanized anti-TLR4 antibody Fab for 90 min. Cells were then washed three times with phosphate-buffered saline (PBS). Cells treated with the humanized anti-TLR4 antibody Fab were stained with the FITC (fluorescein isothiocyanate)-conjugated anti-human IgG at 4 °C away from light for 60 min. The cells were then washed with PBS three times. The cells were then analyzed on a Cytomics FACS Calibur (BD, San Jose, CA, USA).

### 4.4. qPCR (Real Time Quantitative PCR)

Mouse macrophages were cultured at 2.5 × 10^5^ cells/well in 24-well plates and pre-incubated with medium containing humanized anti-TLR4 antibody Fab for 2 h. The cells were then treated with LPS at the different concentrations for 4 h. In order to determine the optimal concentration for later experiments, the concentrations of anti-TLR4 antibody (0.1, 1.5, or 10 µg/mL) and LPS (10, 100, or 1000 ng/mL) were varied. After 4 h of stimulation, the cells were collected and RNA was extracted using a total RNA kit (Omega, Norcross, GA, USA). A quantity amounting to 1 µg total RNA was used in a 10 µL reverse transcription reaction using a PrimeScript RT Master Mix kit (TaKaRa, Shiga, Japan), and the cDNA was then diluted to 40 µL for use as the template in real-time qPCR using a real-time PCR machine (ABI7500; Applied Biosystems, Foster City, CA, USA) with a standard SYBR Green PCR kit (TaKaRa, Kofu, Japan). The reactions were carried out for 40 cycles (94 °C for 5 s, 60 °C for 10 s, and 72 °C for 30 s). All samples were tested in duplicate. The primer sequences used for Q-PCR as described previously [18]: mouse TNF-α, forward 5′-GACGTGGAACTGGCAGAAGAG-3′ and reverse 5′-TTGGTGGTTTGTGAGTGTGAG-3′; mouse IFN-β, forward 5′-CAGCTCCAAGAAAGGACGAAC-3′ and reverse 5′-GGCAGTGTAACTCTTCTGCAT-3′; mouse IL-1, forward 5′-GCAACTGTTCCTGAACTCAACT-3′ and reverse 5′-ATCTTTTGGGGTCCGTCAACT-3′; Mouse IL-6, forward 5′-TAGTCCTTCCTACCCCAATTTCC-3′ and reverse 5′-TTGGTCCTTAGCCACTCCTTC-3′; and mouse β-actin, forward 5′-AGTGTGACGTTGACATCCGT-3′ and reverse 5′-GCAGCTCAGTAACAGTCCGC-3′.

The 2^−∆∆*C*t^ method was used to calculate the relative cytokines of the *TNF*-α, *IFN*-β, *IL*-*1*, and *IL*-*6* gene compared with that of *β-actin*.

### 4.5. Measurement of Cytokine Levels in Culture Supernatants

For cytokine assay, cell culture supernatants were collected after LPS stimulation in the presence or absence of humanized anti-TLR4 antibody Fab. The concentrations of pro-inflammatory cytokines, *i.e.*, TNF-α, IFN-β, IL-1, and IL-6, were determined by ELISA using mouse TNF-α, IL-1, IL-6 (R&D Systems, Minneapolis, MN, USA), and IFN-β ELISA kits (PBL, Piscataway, NJ, USA) according to the manufacturers’ instructions. Briefly, for analysis of TNF-α, IL-1, and IL-6 production, 50 µL of sample diluents plus 50 µL test samples or cytokine standards were added to each well, and plates were incubated at 37 °C for 2 h. The wells were then washed with washing buffer four times. Mouse TNF-α, IL-1, or IL-6 conjugate (100 µL/well) was then added, and plates were further incubated for an additional 2 h at room temperature. After washing the wells four times, 100 µL of substrate solution was added to each well, and the plates were incubated away from light for 20 min. Next, 100 µL stop solution was added to each well, and plates were read at 450 nm. To measure IFN-β production, 100 µL test sample or 100 µL cytokine standard was added to each well, and the plates were incubated at room temperature for 1 h. After washing the wells with wishing buffer three times, antibody solution (100 µL/well) was added, and the plates were further incubated for 1 h at room temperature. After washing three times, horseradish peroxidase (HRP) solution was added, and the plates were then incubated for 1 h at room temperature. Next, 100 μL TMB substrate solution was added, and the plates were incubated away from light for 15 min. Stop solution (100 μL/well) was added to each well, and the signals were read at 450 nm.

### 4.6. Western Blot Analysis

To measure inhibition of NF-κB/MAPK/IRF3, we cultured mouse macrophages at 1 × 10^6^ cells/well in six-well plates in RPMI 1640 medium containing 10% FBS. Cells were pretreated with the humanized anti-TLR4 antibody Fab (1 µg/mL) for 2 h and then treated with LPS (1000 ng/mL) for 0, 15, 30, or 60 min. NF-κB/MAPK/IRF3 inhibition was then detected using Western blot analysis as previously described [19]. Briefly, cells were lysed in RIPA (Radio Immunoprecipitation Assay) buffer supplemented with 1 mM PMSF (Phenylmethanesulfonyl fluoride), protease, and a phosphatase inhibitor mixture. Lysates were sonicated and centrifuged at 12,000× *g* for 10 min at 4 °C. The concentration of proteins was measured with a BCA (Bicinchoninic acid) protein assay kit (Thermo, Waltham, MA, USA). Equal amounts of proteins were loaded on 10% sodium dodecyl sulfate polyacrylamide gels and transferred onto nitrocellulose membranes. The membranes were blocked with 5% non-fat milk or 5% BSA (Bovine serum albumin) in Tris-Buffered Saline and Tween 20 (TBST, 10 mM Tris, pH 7.5, 50 mM NaCl, and 0.1% Tween-20 (Sigma, St. Louis, MO, USA)) for 1 h at 37 °C. The membranes were then marked with primary antibodies (p-p38, p-p65, p-JNK, p-ERK, p-IκB, p-IRF3, p-IKK, or β-actin (1:1000)) diluted in 5% nonfat milk or 5% BSA in TBST for 1 h at room temperature or overnight at 4 °C. Membranes were then washed with TBST three times and incubated with HRP-conjugated goat anti-rabbit or anti-mouse IgG secondary antibodies (Sigma) in 5% non-fat milk or 5% BSA in TBST at room temperature for 1 h. The signal was detected by an enhanced chemiluminescence kit, in accordance with the recommendations of the manufacturer (Millipore Corporation, Billerica, MA, USA).

### 4.7. Statistical Analysis

All experiments were repeated at least three times, and the data were presented as the means ± standard deviations (SDs). Data were evaluated for statistical significance using SPSS 13.0 for Windows. Differences were considered significant when *p* < 0.05.

## 5. Conclusions

Our work demonstrated that our newly developed humanized anti-TLR4 antibody could effectively inhibit the LPS response through TLR4 in mouse macrophages. In future studies, we will examine whether this humanized anti-TLR4 antibody can inhibit the LPS response through TLR4 in other types of immunocytes and in animal models. We expect that this humanized anti-TLR4 antibody may have applications in endotoxemia therapy. One significant limitation of the study is that the expression levels of cytokines have also shown a slight increase in humanized anti-TLR4 antibody Fab without LPS groups. Essentially, this issue should be resolved prior to the clinical usage of humanized anti-TLR4 antibody Fab.
